# Inhibition of Firefly Luciferase by General Anesthetics: Effect on In Vitro and In Vivo Bioluminescence Imaging

**DOI:** 10.1371/journal.pone.0030061

**Published:** 2012-01-10

**Authors:** Marleen Keyaerts, Isabel Remory, Vicky Caveliers, Karine Breckpot, Tomas J. Bos, Jan Poelaert, Axel Bossuyt, Tony Lahoutte

**Affiliations:** 1 In Vivo Cellular and Molecular Imaging (ICMI) Laboratory, Vrije Universiteit Brussel (VUB), Brussels, Belgium; 2 Department of Nuclear Medicine/BEFY, UZ Brussel, Vrije Universiteit Brussel (VUB), Brussels, Belgium; 3 Department of Anesthesiology, UZ Brussel, Vrije Universiteit Brussel (VUB), Brussels, Belgium; 4 Laboratory of Molecular and Cellular Therapy, Department of Immunology and Physiology, Vrije Universiteit Brussel (VUB), Brussels, Belgium; 5 Department of Hematology and Immunology, Vrije Universiteit Brussel (VUB), Brussels, Belgium; MGH, MMS, United States of America

## Abstract

Bioluminescence imaging is routinely performed in anesthetized mice. Often isoflurane anesthesia is used because of its ease of use and fast induction/recovery. However, general anesthetics have been described as important inhibitors of the luciferase enzyme reaction.

**Aim:**

To investigate frequently used mouse anesthetics for their direct effect on the luciferase reaction, both in vitro and in vivo.

**Materials and Methods:**

isoflurane, sevoflurane, desflurane, ketamine, xylazine, medetomidine, pentobarbital and avertin were tested in vitro on luciferase-expressing intact cells, and for non-volatile anesthetics on intact cells and cell lysates. In vivo, isoflurane was compared to unanesthetized animals and different anesthetics. Differences in maximal photon emission and time-to-peak photon emission were analyzed.

**Results:**

All volatile anesthetics showed a clear inhibitory effect on the luciferase activity of 50% at physiological concentrations. Avertin had a stronger inhibitory effect of 80%. For ketamine and xylazine, increased photon emission was observed in intact cells, but this was not present in cell lysate assays, and was most likely due to cell toxicity and increased cell membrane permeability. In vivo, the highest signal intensities were measured in unanesthetized mice and pentobarbital anesthetized mice, followed by avertin. Isoflurane and ketamine/medetomidine anesthetized mice showed the lowest photon emission (40% of unanesthetized), with significantly longer time-to-peak than unanesthetized, pentobarbital or avertin-anesthetized mice. We conclude that, although strong inhibitory effects of anesthetics are present in vitro, their effect on in vivo BLI quantification is mainly due to their hemodynamic effects on mice and only to a lesser extent due to the direct inhibitory effect.

## Introduction

Bioluminescence imaging (BLI) has emerged over the last decades as a non-invasive assessment of a molecular target. A luciferase reporter gene is expressed in cells of interest and the enzymatic turnover of luciferase after administration of its substrate allows the readout of the reporter gene activity. The signal intensity reflects the strength or changes of a molecular target in a quantitative way. *In vivo*, many other parameters can influence the BLI readout, such as the administration route and protein binding of the substrates, overlying tissues characteristics and membrane pumps that can remove substrates out of the cell [1], [2], [3], [4], [5], [6].

During BLI, anesthesia of mice and rats is performed to reduce changes is signal intensity due to movement of the animal. However, a direct inhibitory effect of some general anesthetics on the luciferase enzyme has been described in literature. Already in 1976, the inhibitory effect of local anesthetics on firefly luciferase (Fluc) was reported [7]. Since then, the interaction of Fluc with anesthetics has been considered the best-characterized model system for studying anesthetic–protein interactions [8], [9], [10]. This research revealed that several local and general anesthetics have an inhibitory effect on the luciferase activity by direct binding to the enzyme, at concentrations similar to those that induce general anesthesia in animals [11], [12]. Whether the binding of the inhibitor is substrate-competitive or non-competitive has been long debated and has not reached a consensus thus far. Recently, the hypothesis that some anesthetics can not only bind at the D-luciferin binding site, but also to a domain that regulates the opening and closing of the enzymatic pocket has been proposed. Binding of anesthetics to this domain results in closing of the enzymatic cleft and it therefore inhibits the binding of D-luciferin [13].

Besides a potential direct inhibitory effect of the luciferase reaction, anesthetics influence the cardiovascular condition of the test animal, thereby potentially altering the delivery of the substrate to the cells of interest and thus the BLI signal intensity [14]. The effect of general anesthetics on BLI *in vivo* has so far only been investigated in a small comparative study by Cui et al., in which isoflurane and avertin led to lower BLI signals compared to ketamine/xylazine [15]. We hypothesize, based on these previously reported direct and indirect effects of anesthetics, that general anesthetics, used during BLI, will affect the intensity and kinetics of the bioluminescent signal *in vivo*.

The aim of this study was to investigate the effect of different currently used mouse anesthetics on a Fluc-expressing cell line, both *in vitro* and *in vivo*. We here show that, although strong luciferase-inhibiting effects of anesthetics are present *in vitro* for volatile agents and avertin, their effect on *in vivo* BLI quantification is mainly due to their hemodynamic effect on the mice and only to a lesser extent due to a direct effect on the luciferase enzyme itself. For high sensitivity, unanesthetized BLI or BLI using pentobarbital are the most suited, followed by avertin. Isoflurane, although very user friendly, as well as ketamine/medetomidine anesthesia reduce sensitivity. Thorough standardization of the anesthesia, both in dosage and time between induction and substrate injection, should improve the reproducibility of the technique.

## Materials and Methods

### Ethics statement

The study protocol was approved by the Institutional Animal Care and Use Committee of Vrije Universiteit Brussel, permit number 10-272-3, and National Institutes of Health principles of laboratory animal care (NIH publication 86-23, revised 1995) were followed.

### Cell lines

The commercial vector pGL4.10 (Promega, Madison, WI, USA), encoding Firefly luciferase (Fluc) and the thermostable red-shifted Firefly luciferase (Ppy RE–TS), kindly provided by Branchini et al. [16], were constitutively expressed in a R1M rhabdomyosarcoma cell line (R1M-Fluc) and 293T cell line (293T-Fluc) respectively, as was previously described [4], [17]. R1M-Fluc cells were grown in Minimal Essential Medium (MEM) with 10% fetal bovine serum (FBS), 1% non-essential amino acids, 100 U/mL penicillin, 100 µg/mL streptomycin and 0.13 µg/mL fungizone (all from Invitrogen, Paisley, UK). 293T-Fluc cells were grown in Dulbecco's modified Eagle medium (DMEM) supplemented with 100 U/mL penicillin, 100 µg/mL streptomycin, 2 mM L-glutamine (all from Lonza, Verviers, Belgium) and 10% FBS (Biochrom AG, Berlin, Germany).

### Substrates

D-luciferin (Promega, Madison, WI, USA) was diluted in phosphate buffered saline (PBS) to obtain a stock solution of 30 mg/ml, after which the solution was sterilized using a 0.22 µm filter for *in vivo* use.

### Anesthetics

For all anesthetics, a literature search was performed to document the conventional *in vivo* doses for mice. For volatile anesthetics, these doses are reported as minimum alveolar concentration or MAC. MAC is the concentration of a volatile anesthetic that is needed to prevent movement in 50% of subjects in response to pain stimulus [18]. A lower MAC value represents a more potent inhalation anesthetic. For surgical procedures, a concentration of 1.2–1.5× MAC is usually used. Table 1 shows the corresponding MAC values per volatile anesthetic [19], [20], [21], [22].

**Table 1:** Physiological anesthetic ranges for volatile anesthetics.

Anesthetic agent	MAC	*in vivo* dose range	references
Isoflurane	1.2–1.8%	1.6–2.3%	[32], [33]
Sevoflurane	2.2–2.9%	2.9–3.8%	[34], [35]
Desflurane	6.5–9.1%	8.5–11.8%	[33]

MAC = minimum alveolar concentration.

For injectable anesthetics, doses are expressed in mg/kg. Conventionally used doses are shown in Table 2 [23], [24], [25]. For *in vitro* use, we converted these doses to mg/L, assuming a homogenous distribution of the anesthetic in the whole body and a mass density of mice of 1 L/kg. The *in vivo* dose range is indicated in the figures using grey intervals.

**Table 2:** Physiological anesthetic ranges for injectable anesthetics.

Anesthetic agent	*in vivo* dose range	references
Ketamine	18–200 mg/kg	[36], [37], IACUC
Xylazine	5–20 mg/kg	[37], IACUC
Pentobarbital	40–70 mg/kg	[38], IACUC
Medetomidine	0.5–1.0 mg/kg	[36], IACUC
Avertin	200–400 mg/kg	IACUC

IACUC = Institutional Animal Care and Use Committee.

Following stock solutions of anesthetics were used: isoflurane (1-chloro-2,2,2-trifluoroethyl-difluoromethyl ether, Forene, Abbott, England); sevoflurane (fluoromethyl 2-2 difluoro 1-trifluoromethyl vinyl ether, Sevorane, Abbott, England), desflurane (2,2,2-trifluoro-1-fluoroethyl-difluoromethyl ether, Suprane, Baxter, Belgium); 100 mg/ml ketamine hydrochloride (Ketamine 1000 CEVA, CEVA Santé Animale); 20 mg/mL xylazine (Rompun 2%, Bayer, Belgium); 60 mg/ml natrium pentobarbital (Nembutal, CEVA Santé Animale, Belgium); 1 mg/ml medetomidine hydrochloride (Medetor, Virbac, Belgium). Avertin (2,2,2-tribromoethanol) was purchased from Sigma-Aldrich (Germany) and 5 g was dissolved in 2-methyl-2-butanol (Sigma-Aldrich) to obtain a stock concentration of 1.6 g/mL by stirring overnight at room temperature protected from light. This stock solution was further dissolved in 0.9% NaCl to obtain a final concentration of 20 mg/mL (overnight stirring at room temperature protected from light), after which the solution was sterilized using a 0.22 µm filter, kept at 4°C protected from light and was used within 1 month after dissolution.

### In vitro intact cell BLI measurements

For *in vitro* measurements, R1M-Fluc cells were plated in normal growth medium in 25 cm^2^ culture flasks (1×10^6^ cells/flask) or 24-well plates (75×10^3^ cells/well). After overnight incubation at 37°C and 5% CO_2_ to allow adherence, cells were preincubated with the anesthetic during 10 minutes. For volatile anesthesia, 25 cm^2^ culture flasks were used. A continuous flow of oxygen with the appropriate percentage of inhalation anesthetic above the media was created. The following volatile anesthetics were used (concentrations indicated in brackets): isoflurane (0.0%, 0.5%, 1.0%, 1.5%, 2.0%, 2.5%, 3.0%); sevoflurane (0.0%, 0.5%, 1.0%, 1.5%, 2.0%, 2.5%, 3.5%, 5.0%); desflurane (0.0%, 1.0%, 3.0%, 6.0%, 9.0%, 12%, 18%, 21%).

For injectable anesthetics, 24-well plates were used. The cell medium was replaced by fresh cell medium containing the appropriate concentration of the anesthetic. The following injectable anesthetics were used (final concentrations indicated in parentheses): ketamine (0.0 mg/L, 20 mg/L, 100 mg/L, 200 mg/L, 400 mg/L); xylazine (0.0 mg/L, 4.0 mg/L, 10 mg/L, 20 mg/L, 40 mg/L); pentobarbital (0.0 mg/L, 14 mg/L, 50 mg/L, 70 mg/L, 140 mg/L); medetomidine (0.0 mg/L, 0.10 mg/L, 0.50 mg/L, 1.0 mg/L, 2.0 mg/L); avertin (0.0 mg/L, 48 mg/L, 120 mg/L, 240 mg/L, 480 mg/L).

BLI measurements were performed as described in [4]. Measurements were started immediately after the addition of the substrate (final concentration of D-luciferin: 0.15 mg/ml), in the presence of the anesthetic, using a Photo Imager camera (Biospace, France) that allows list mode acquisition. Experiments were performed in triplicate (inhalation anesthetics) or quadruplicate (injectable anesthetics). Identical circular Regions of Interest (ROI) were drawn over the wells and their Photon emission (PE) using 5 sec intervals was analyzed. The maximum photon emission (PE_max_) of the dynamic profile of 10 minutes containing the peak photon emission was derived using the 95^th^ percentile. Per condition, an average of 9 (inhalation anesthesia) or 12 (injectable anesthesia) samples (3 experiments performed in triplicate/quadruplicate) is expressed as % of control ± standard deviation (SD).

pH of anesthetic solutions in cell media was assessed using pH-indicator strips pH 4.0–7.0 and 6.5–10.0 (Merck, Darmstadt, Germany).

### In vitro cell lysate BLI measurements

To analyze the effect of injectable anesthetics on the luciferase reaction in the absence of cell membranes, which might interfere with the diffusion of the substrate into the cells, results of the intact cell measurements were compared to those obtained on cell lysates. A commercially available Luciferase Assay Reagent (promega) was used, which contains all necessary substrates for the reaction in excess [26].

In a white 96-well plate, 1×10^4^ R1M-Fluc cells were added to 50 µL of Reporter Lysis Buffer (Promega) per well, and a single freeze-thaw cycle was performed. Per well, 10 µL of injectable anesthetics were added at appropriate concentrations to reach final concentrations, identical to the concentrations used in the intact cell measurements. Measurements were performed using a glomax-96 microplate luminometer with auto-injector system (Promega). BLI intensity was measured at baseline (before addition), and with a 10 s delay after the addition of 50 µL of Luciferase Assay Reagent, using the automated injector. The integration time was set at 5 s for all measurements. For further analysis, values after addition of the Luciferase Assay Reagent were corrected by subtracting the baseline signal. Per condition, an average of 12 samples (3 experiments performed in quadruplicate) is expressed as % of control ± SD.

### Mice

Male athymic nude (Hsd:Athymic Nude-Foxn1^nu^) mice were purchased from Harlan Laboratories (Boxmeer, The Netherlands) and were between 5 and 11 weeks old before initiation of experiments. Mice were kept in individually ventilated cages (Techniplast, Buguggiate, Italy) on sawdust on a 12-h day/night cycle with water and food ad libitum.

### In vivo BLI

293T-Fluc cells (1×10^6^ cells in matrigel) were subcutaneously injected in mice at day 0 during a short anesthesia with isoflurane. Mice were imaged at day 3, 5 and 7 post cell injection, alternating between isoflurane anesthesia (2%) and the test anesthetic with a cross over design, so that half of the mice had twice isoflurane and half of the mice had twice the test anesthetic [3]. For the final signal intensity, the average of day 3 and day 7 was compared to day 5, to correct for cell growth between the different imaging sessions. Four anesthetic conditions were compared to isoflurane in 4 different groups of mice: no anesthesia (n = 10); ketamine 37.5 mg/kg/medetomidine 0.5 mg/kg (n = 12) [27]; pentobarbital 60 mg/kg (n = 14) [28]; avertin 400 mg/kg (n = 12) [29]. Injectable anesthetics were injected intraperitoneally without induction anesthesia. After a brief induction with 2% isoflurane anesthesia, or when mice were non-reactive after injection of the test anesthetic, D-luciferin was injected intravenously at a weight-dependent substrate dose of 150 mg/kg.

Immediately after substrate administration, mice were imaged using the Photon Imager (Biospace, France). For unanesthetized imaging, the photon imager was equipped with the *in actio* module (Biospace), which simultaneously records both bioluminescence signal and a bright field video of the animal under infrared illumination for co-registration (Fig S2 and video S1). Mice were placed inside the BLI camera on a heated platform to maintain a physiological body temperature. Using a nose cone, volatile anesthetics in O_2_ or 100% O_2_ were administered during the entire acquisition. The photon emission was measured dynamically during 45 min, except for the isoflurane/unanesthetized study, for which the acquisition duration was 50 min. For image analysis an elliptical ROI was drawn over the mouse bearing the 293T-Fluc cells. The surface area of the ROI was kept constant. A time activity curve for every acquisition was obtained by analyzing images in 5 s intervals. For the calculation of the PE_max_ from the tumor, the 95^th^ percentile of 5 s intervals was used, as was described previously [4]. All anesthetics and the unanesthetized imaging were compared to the conventionally used isoflurane anesthesia (2%) and are expressed as % of this control condition. For the calculation of the time-to-peak, the same acquisition data were analyzed using 1 min intervals, and the time point containing the highest photon emission was defined as time-to-peak. For the isoflurane/unanesthetized study, the area under the curve (AUC) was calculated over 50 min by making the sum of all 5 s intervals and expressed as % of isoflurane.

### Statistical analysis

All data are represented as mean ± SD. Statistical analysis was carried out in Prism 5.0 (GraphPad software Inc.). Unpaired t test was used to compare means of 2 groups for *in vitro* experiments. *In vivo* BLI intensity data were analyzed using two-tailed Wilcoxon signed rank tests for each test anesthetic in comparison to isoflurane (4 groups of mice). Time-to-peak of different anesthetics was compared in all the groups of mice taken together using a Kruskal-Wallis test and a Dunn's multiple comparison test. A p value<0.05 was considered to be significant.

## Results

For all anesthetics, the effect on in vitro luciferase activity of physiological in vivo dose ranges is summarized in Table 3.

**Table 3:** In vitro BLI at physiological anesthetic range.

Anesthetic agent	*In vivo* dose range	average % of untreated cells	
		intact cells	cell lysate
Isoflurane	1.6–2.3%	65.8–50.4%	
Sevoflurane	2.9–3.8%	57.0–51.3%	
Desflurane	8.5–11.8%	65.0–56.8%	
Ketamine	18–200 mg/kg	114–164%	101–102%
Xylazine	5–20 mg/kg	107–147%	96.4–98.8%
Pentobarbital	40–70 mg/kg	104–101%	106–104%
Medetomidine	0.5–1.0 mg/kg	103–109%	97.3–98.5%
Avertin	200–400 mg/kg	24.1–15.6%	58.3–38.8%

### Volatile anesthetics in vitro

**Figure 1:**
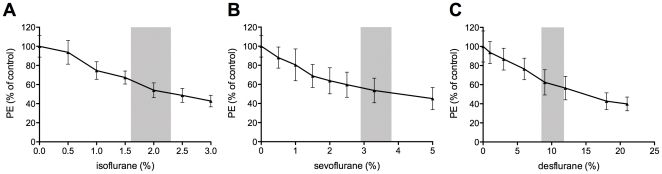
Effect of volatile anesthetics on BLI signal. The effect on photon emission (PE) of isoflurane (**A**), sevoflurane (**B**) and desflurane (**C**) anesthetics is expressed relative to control samples, containing 100% oxygen without inhalation anesthetic. Intact cells were used. Grey intervals in the curves indicate the in vivo dose ranges.

In a first part, we sought to investigate if a direct inhibitory effect, as was already described for halothane on pure luciferase enzyme, could be detected for regularly used volatile mouse anesthetics in intact cells as well [30]. Fluc expressing R1M cells were allowed to adhere in small culture flasks overnight. They were incubated with a continuous flow of a mixture of oxygen and the volatile anesthetic above the cell media during 10 min, after which D-luciferin was added and photon emission (PE) was measured. A preliminary study showed no difference in bioluminescent signal obtained from cells using incubation with 100%, 95%, 50% or 25% O_2_ (Fig. S1). At the end of the experiment, cells were analyzed microscopically, which showed no change in morphology of the cells. Results for the three volatile anesthetics tested are shown in Figure 1. For isoflurane, the most often used volatile anesthetic in mice, there is an important decrease in the bioluminescent signal with increasing amounts of isoflurane (Fig. 1A), indicating an important and dose-dependent inhibitory effect. Within the physiological dose range for isoflurane, signal intensities drop to 50.4–65.8% of control values. Sevoflurane showed the most pronounced inhibitory effect, with average signals of 57.0–51.3% at 1.3× MAC (Fig. 1B). Results for desflurane are comparable to isoflurane, with a drop in signal intensity to 56.8–65.0% at 1.3× MAC (Fig. 1C).

### Injectable anesthetics in vitro

Since inhibitory effects were also described for injectable anesthetics such as lidocaine, we further wanted to analyze if regularly used mouse anesthetics for injection showed a direct inhibitory effect on the luciferase reaction [31]. Cells were plated in well plates and incubated in the presence of the anesthetic during 10 min, after which the substrate D-luciferin was added and bioluminescent signals were quantified. Since for some anesthetics, an increase rather than a decrease in BLI signal was detected, the results were further supplemented with experiments using cell lysates and the commercially available luciferase assay kits containing all the substrates needed for the bioluminescent reaction, to evaluate if a change in diffusion through the cell membrane was responsible for the observed increase in BLI signal. Cells were also evaluated morphologically by light microscopy at the end of BLI. R1M rhabdomyosarcoma cells grow adherent and have a fibroblast-like spindle shape in normal conditions.

**Figure 2:**
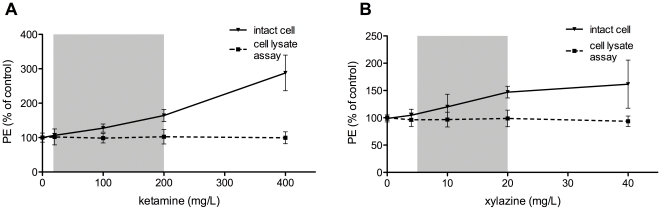
Effect of ketamine and xylazine on BLI signal of intact cells and cell lysates. The effect on photon emission (PE) of the anesthetics ketamine (**A**) and xylazine (**B**) are shown using intact cells (full line) and cell lysates (dotted line). All data are represented as mean % of control (absence of anesthetic) ± SD. Grey intervals in the curves indicate the concentrations used *in vivo*, as reported in literature for mice.

The effects of ketamine and xylazine on PE are shown in Figure 2. An increase in PE is seen with increasing amounts of the anesthetic, up to 288% for the highest used dose of ketamine. However, this effect is abrogated when cell lysates are used instead of intact cells, indicating that the increase in bioluminescent signal is due to a higher cell membrane permeability. When evaluating the intact cells morphologically after BLI, signs of cell toxicity, such as cell rounding and detachment, were noticed. This effect was detected for 200 and to a stronger extent for 400 mg/L ketamine. Below 200 mg/L ketamine, no changes were seen in cell morphology. For xylazine, this effect was less pronounced and only present at the highest concentration. These changes in cell morphology indicate a toxic effect on the cells.

**Figure 3:**
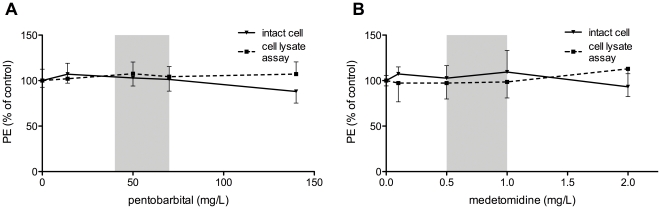
Effect of pentobarbital and medetomidine on BLI signal of intact cells and cell lysates. The effect on photon emission (PE) of the anesthetics pentobarbital (**A**) and medetomidine (**B**) are shown using intact cells (full line) and cell lysates (dotted line). All data are represented as mean % of control (absence of anesthetic) ± SD. Grey intervals in the curves indicate the concentrations used *in vivo*, as reported in literature for mice.

For pentobarbital and medetomidine, there is no significant effect in the *in vivo* dose ranges (Fig. 3). Figure 3A shows a decrease in PE for high doses of pentobarbital in the intact cell assay, but not for cell lysates. This indicates a possibly weak inhibitory effect on the luciferase enzyme, only at supraphysiological concentrations, that is no longer present in the cell lysate assay, since then higher doses of the substrates are used. There were no changes in cell morphology at the end of the experiment.

**Figure 4:**
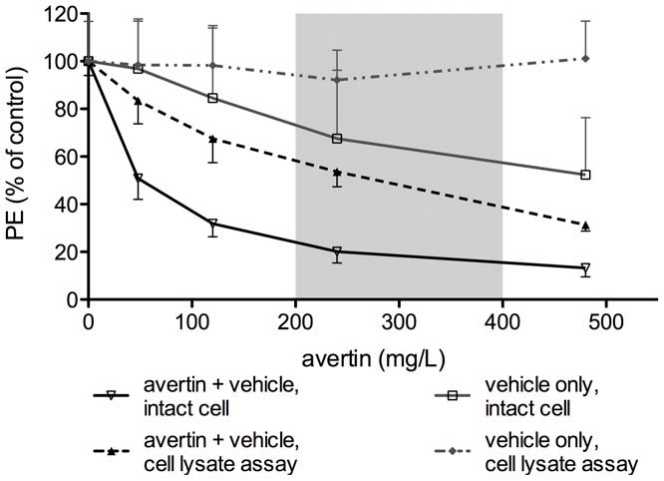
Effect of avertin and its vehicle on BLI signal of intact cells and cell lysates. The effect on photon emission (PE) of the anesthetic avertin (black) and its vehicle 2-methyl-2-butanol (grey) are shown using intact cells (full line) and cell lysates (dotted line). All data are represented as mean % of control (absence of anesthetic) ± SD. The grey interval in the curve indicates the concentration used *in vivo*, as reported in literature for mice.

To test the effect of avertin that is dissolved in the vehicle 2-methyl-2-methanol, both the vehicle and the combination with avertin were tested. As shown in Figure 4, there is a very strong effect in intact cells, which is mainly due to avertin itself, but the effect is also to a minor extent present for the vehicle alone. When cell lysates are used, the effect is no longer present for the vehicle, and only moderately for vehicle+avertin, suggesting that the higher amounts of substrates in this assay can partly overcome the inhibitory effect. At microscopic evaluation, no changes in cell morphology were observed at the end of the experiment.

To exclude any effect of a change in pH due to the addition of anesthetics to the cell media, pH of the highest anesthetic concentration was compared to media without anesthesia and no change in pH above 0.5 was detected.

The inhibitory effect of isoflurane and avertin was also quantified in 293T-Fluc cells, which were used in *in vivo* experiments, with an inhibitory effect comparable to that observed in R1M-Fluc cells (data not shown).

### Reversibility of inhibitory effect by higher substrate concentration

**Figure 5:**
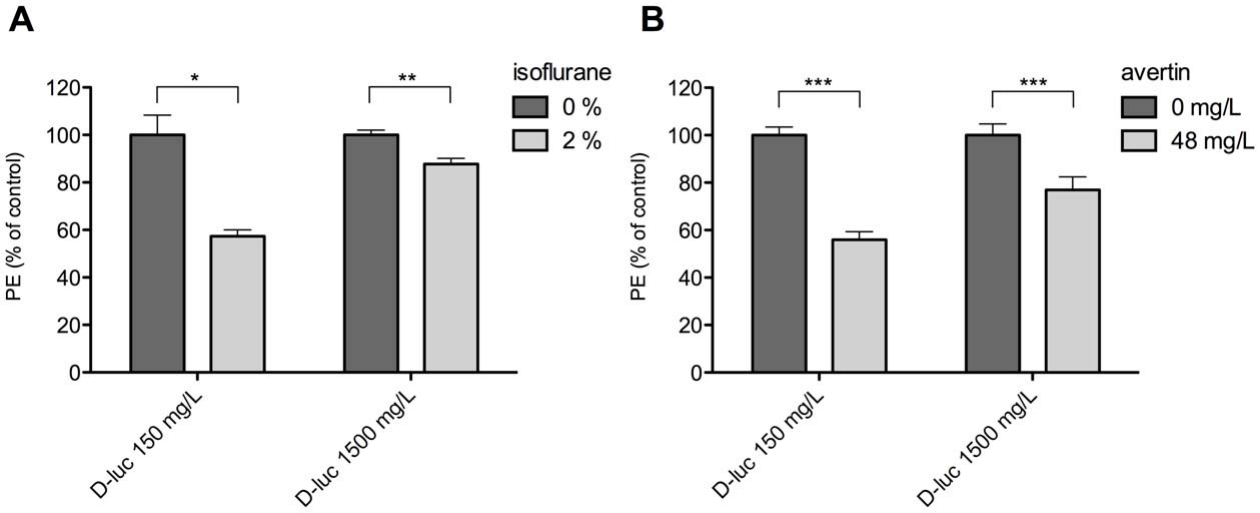
Reversibility of photon emission inhibition by high substrate concentration. Photon emission and its inhibition by isoflurane (**A**) and avertin (**B**) was examined at normal concentration of D-luciferin (D-luc 150 mg/L) and a 10× higher concentration (D-luc 1500 mg/L) to assess the reversibility of the inhibition. * p<0.05, ** p<0.01, *** p<0.0001.

In literature, there has been some controversy about the underlying mechanism of luciferase inhibition by anesthetics, being (non-)competitive or (non-)reversible. To further investigate this question, we examined the effect of increased substrate concentrations on the inhibitory effect of anesthetics. The intact cell assay was repeated with 10× higher doses of D-luciferin for both isoflurane and avertin, at doses at which PE was reduced to around 50% of control values (2% isoflurane and 48 mg/L avertin). Figure 5 shows that the inhibition can partly be overcome by using D-luciferin at 1500 mg/L instead of 150 mg/L, to 88% and 77% of control values for isoflurane and avertin respectively. This result indicates that the effect is reversible but non-competitive, since the reversibility is not complete.

### In vivo comparison of anesthetics

To compare the effect of anesthesia on BLI *in vivo*, the bioluminescent signal obtained using different anesthetics was intra-individually compared to isoflurane, serving as the standard. Isoflurane anesthesia was also compared to unanesthetized mice. Isoflurane was chosen as the standard because bioluminescence cameras are all equipped with gas anesthesia inlet and outlet ports, resulting in the majority of BLI measurements being performed using isoflurane anesthesia. In unanesthetized animals, variations in the signal over time was noted due to movement of the animal, making this method less suited as the standard. An example of the bioluminescent signal in an unanesthetized mouse is added in the supplementary data (Fig. S2 and video S1).

**Figure 6:**
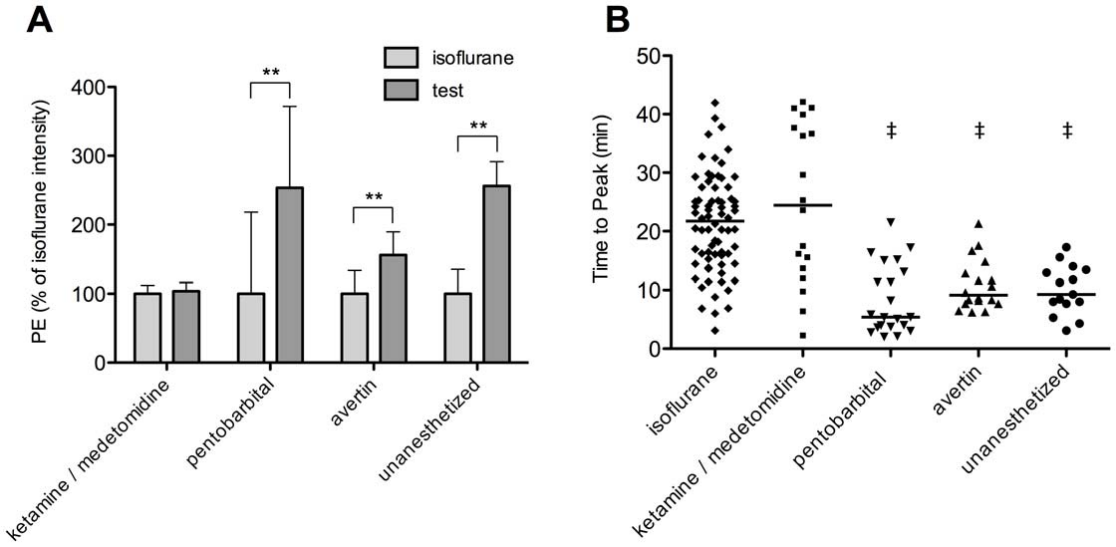
In vivo comparison of anesthetics. (**A**) represents the maximum BLI intensity obtained from the same mice anesthetized with isoflurane or the test anesthetic mentioned below the curve. Values were normalized to isoflurane signal intensities. (**B**) shows a scatter plot of the time to the peak signal intensity for the different anesthetics. Each data point represents a unique bioluminescent acquisition from the in vivo comparison of anesthetics study. Lines indicate the mean. There was a significantly later peak for ketamine/medetomidine and isoflurane as compared to avertin, pentobarbital and unanesthetized animals. ** p<0.01 between indicated conditions; *‡ p<0.05 compared to both isoflurane and ketamine/medetomidine*.

Figure 6A displays the differences in peak bioluminescent signal using different or no anesthetics, normalized to the obtained value for isoflurane (100%). It clearly shows that unanesthetized animals as well as pentobarbital-anesthetized mice have a significantly higher peak signal than using isoflurane anesthesia (256%, p = 0.002; 254%, p = 0.009 respectively). This effect is less pronounced for avertin anesthesia (156%, p = 0.009). The combination of ketamine/medetomidine anesthesia gives a signal intensity that is comparable to isoflurane anesthesia (104%, ns). This intensity is on average 40% of unanesthetized and pentobarbital-anesthetized signal intensities, indicating a clear negative effect of these anesthetic conditions on the bioluminescent peak signal.

Not only the intensity of the signal was evaluated in these mice, but also the time that elapsed between the injection of the substrate D-luciferin and the time of the maximal signal intensity, termed “time to peak”. Figure 6B reflects the time of the peak for all performed acquisitions according to the used anesthetic condition. There was a significantly later peak for ketamine/medetomidine and isoflurane as compared to avertin, pentobarbital and unanesthetized animals.

**Figure 7:**
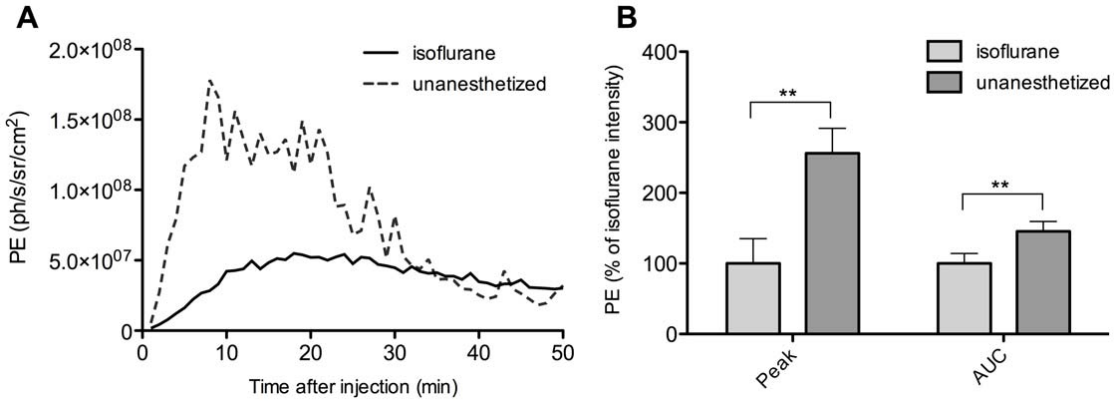
Comparison of peak signal and AUC of the signal for isoflurane-anesthetized and unanesthetized mice. (**A**) shows a representative example of the time profile obtained using isoflurane anesthesia (full line) and without anesthesia (dotted line). (**B**) shows the average peak signal and AUC of the signal for the same mice, anesthetized with isoflurane or without anesthesia. ** p<0.01 between indicated conditions. AUC area under the curve.

Although there was a clear difference in time to peak for different anesthetic conditions, the overall amount of light produced, calculated by the AUC of the time activity curve, might still be the same. To investigate this hypothesis, peak signal intensities were compared to AUC values for mice imaged with isoflurane anesthesia and without anesthesia. Representative time activity curves of 1 mouse are shown in Figure 7A, showing a fast rise and a more rapid decline in the BLI signal for the unanesthetized acquisition compared with 2% isoflurane. In Figure 7B, the average of all mice for peak intensity is compared to total PE, calculated as the AUC during 50 minutes, for both anesthetic conditions. The difference in BLI signal between the two conditions is indeed less pronounced when AUC is calculated (146% for AUC compared to 256% for peak photon emission), but it is still significantly higher in unanesthetized animals (p = 0.002), showing that not only the peak signal but also the total amount of signal produced is different in these two anesthetic conditions. For isoflurane and ketamine/medetomidine, there was no significant difference in the AUC values (p = 0.46, data not shown).

Using avertin and pentobarbital, mice usually woke up before the end of the acquisition and started moving around inside the camera between 30 and 40 min after tracer injection. This finding does not allow a correct calculation of the AUC for avertin and pentobarbital. When pentobarbital was used, mice often remained responsive to pain stimuli at the time of D-luciferin injection.

## Discussion

Bioluminescence imaging is a recently improved imaging technique that allows *in vivo* quantitative assessment of the reporter gene activity in small animals such as mice and rats, after the administration of its substrate. It allows easy transition from *in vitro* reporter assay systems using the luciferase gene to analysis of these same phenomena in living mice. Mice are anesthetized during the imaging to avoid movement artifacts within the acquired data. Often volatile anesthetics are chosen because of their ease of use. Isoflurane anesthesia units are now routinely provided together with most BLI cameras on the market.

Many anesthetics, such as halothane and lidocaine, have however been proven to exert a direct inhibitory effect upon the luciferase enzyme by binding to the protein and changing the catalytic velocity of the enzyme, either in a competitive or non-competitive way [11]. We therefore wanted to investigate and characterize the possible inhibitory effect of different types of volatile anesthetics as well as injectable anesthetics that are frequently used in rodents. In a second part, we wanted to correlate these *in vitro* findings to changes in signal intensities and kinetics in mice using different anesthetic conditions.

For volatile anesthetics, the results clearly show a dose-dependent inhibitory effect on the bioluminescent reaction of about 50% for all three agents, making neither sevoflurane nor desflurane better alternatives for the frequently used isoflurane. For the injectables ketamine and xylazine, we found the opposite effect, with a dose-dependent increase in bioluminescent signal, which was associated with a microscopic change in cell morphology pointing towards a toxic effect of these agents. We therefore hypothesized that the toxic effect was changing the cell membrane permeability for the substrate D-luciferin, leading to higher intracellular substrate concentrations and thereby a higher bioluminescent signal. This was confirmed by the abrogation of the effect in the absence of the cell membrane barrier, using cell lysate assays. The injectable avertin caused a powerful inhibition of the luciferase reaction of about 80% at physiological concentrations in intact cells. The effect was less pronounced but still clear in the cell lysate assays, most likely because of the higher substrate concentrations in the cell lysate assay. For pentobarbital and medetomidine, no effect on the luciferase reaction was detected at physiological concentrations for both intact cell measurements and cell lysate assays.

We further investigated the reversibility of the inhibition of isoflurane and avertin by challenging the inhibition with a 10-times higher substrate dose. This showed a partially reversible inhibition for both compounds, pointing towards a mixed-type inhibition. This type of luciferase inhibition has been explained by Szarecka et al., as a combination of competitive binding at the substrate binding sites as well as a non-competitive binding at a hinging region of the luciferase enzyme, thereby altering the accessibility of the enzymatic pocket for its substrates [13]. Higher doses of substrate can result in an increased chance to bind within the enzymatic pocket, but will never reach the turnover that is obtained in het absence of the mixed-type inhibitor.

Of course, the most important question is to what extent these inhibitors can influence *in vivo* BLI quantification. In *in vivo* settings, not only a direct effect can be of influence, but also the effect of the anesthetic agent on the cardiovascular condition of the mouse is likely to change the delivery of the substrate to the luciferase-expressing tissue. We therefore compared isoflurane, the most frequently used anesthetic for *in vivo* BLI, to different anesthetic combinations as well as to BLI in unanesthetized mice. The latter was possible by using a dedicated system that allows the simultaneous recording of both bioluminescence signal and a bright field video of the animal under infrared illumination for co-registration (Fig. S2 and video S1). Movement of the mouse however causes changes in the overlying tissues, resulting in fluctuation in the observed signal over time, as can be seen in the curve of Fig. 7A.

When comparing the maximal signal intensities observed using different anesthetic conditions, unanesthetized and pentobarbital anesthetized BLI result in the highest photon emission. Avertin resulted in a lower maximal photon emission than unanesthetized or pentobarbital anesthetized BLI, but still significantly higher than the intensities obtained using isoflurane and ketamine/medetomidine. The majority of these results do not correspond to the inhibition that was measured *in vitro*, where the lowest intensities were obtained for avertin, and no negative effect was observed for ketamine or medetomidine. Only for isoflurane, the inhibition detected *in vitro* correlates well with low *in vivo* signal intensities. Other factors than merely the direct inhibitory effect must therefore play an important role in *in vivo* BLI.

When comparing the maximal *in vivo* signal intensities to the time to peak, high peak values are associated with short time to peak values, indicating that a fast biodistribution of the substrate leads to higher bioluminescent signals. This finding suggests that cardiovascular changes, caused by the anesthetics, induce differences in substrate biodistribution, which alters both maximal signal intensity as well as signal kinetics. A similar observation was made by Cui et al., where ketamine/xylazine resulted in the highest peak photon emission and shortest time to peak. Contrary to our findings, they report equally low values for avertin-anesthetized and isoflurane-anesthetized mice, which might be explained by the lower anesthetic doses that were used.

Pentobarbital and avertin anesthesia resulted in shorter anesthesia duration, with waking up of the mice after 30–40 min. These conditions gave the highest signal intensities, for pentobarbital comparable to unanesthetized mice and for avertin to about 2/3 of unanesthetized animals. In literature, avertin has been named as one of the most suitable anesthetics for functional cardiac assessment since it decreases heart rate to a much lesser extent than ketamine/xylazine anesthesia [20], [22]. Other anesthetics with limited effect on heart rate and cardiac output are isoflurane and to a lesser extent pentobarbital [19], [21], [23], [25]. Among these 3 anesthetics, the lowest BLI signal intensities were obtained using isoflurane and avertin, which suggests that a direct inhibitory effect on the enzymatic reaction could also be partly responsible for their lower BLI intensity compared to unanesthetized and pentobarbital-anesthetized mice. This however remains uncertain since we do not possess information about the cardiovascular condition and tissue perfusion in our mouse study.

Ketamine/medetomidine anesthesia, as well as isoflurane, provided the lowest signal intensities. The combination of ketamine with another alpha2-agonist, xylazine, induces a strong reduction in heart rate and therefore cardiac output, as was already shown in multiple mouse studies [19], [21], [23], [24]. Data are less abundant for the longer acting ketamine/medetomidine combination, but effects are expected to be comparable to ketamine/xylazine [14]. This strong reduction in cardiac output most likely causes a decreased D-luciferin delivery to the luciferase-expressing cells, resulting in lower signal intensities and slower kinetics.

Besides the cardiovascular effect, another factor that might explain the differences observed between *in vitro* and *in vivo* results is the dilution and distribution of the anesthetics. We assumed a perfectly homogenous distribution of the anesthetics in mice, but given their lipophilicity, concentrations will be higher in fatty tissues, such as brain and fat, and lower at the subcutaneous luciferase expression site.

For isoflurane-anesthetized and unanesthetized animals, we not only compared maximum BLI signal but also looked at total photon emission by calculating AUC. Total photon emission was still significantly lower in the isoflurane group, although the difference was smaller. This finding might suggest that AUC is a better parameter to assess luciferase expression levels than peak photon emission, but given the prolonged acquisition times necessary to obtain this value, in our case up to 50 min, it is unlikely this will be the parameter of choice for future experiments.

To achieve maximal sensitivity in *in vivo* BLI, imaging unanesthetized mice is preferable. Disadvantages however include movement artifacts and changes in attenuation by the overlying tissues, causing fluctuations in the intensity that is measured. Moreover, sources at the lateral or ventral side of the mouse will be difficult to image because of the important amount of overlying tissues. The latter however can be overcome by using a 3D dynamic camera, allowing a multiple-view dynamic imaging of a moving mouse. Other sensitive options include pentobarbital and to a lesser extent avertin anesthesia. Isoflurane, although very user-friendly, as well as ketamine/medetomidine are less suited to achieve high sensitivity for *in vivo* BLI. Since cardiovascular effects of ketamine/xylazine are expected to be similar to those of ketamine/medetomidine, it does not hold promise as a good candidate either.

Once the anesthetic approach has been chosen, a thoroughly standardized imaging protocol, including bodyweight-adapted anesthetic dose and a fixed time interval between the induction of the anesthesia and time of substrate injection, should improve reproducibility of the method, by reducing the interfering effects of the anesthesia on the signal intensity.

We conclude that, although strong luciferase-inhibiting effects of anesthetics are present *in vitro* for volatile agents and avertin, their effect on *in vivo* BLI quantification is mainly due to their hemodynamic effect on the mice and only to a lesser extent due to a direct effect on the luciferase enzyme itself. For high sensitivity, unanesthetized BLI or BLI using pentobarbital are the most suited, followed by avertin. Isoflurane, although very user friendly, as well as ketamine/medetomidine anesthesia reduce sensitivity. Thorough standardization of the anesthesia, both in dosage and time between induction and substrate injection, should improve the reproducibility of the technique.

## Supporting Information

Figure S1 **Comparison of BLI intensity using different fractions of oxygen.** 1×10^6^ R1M-Fluc cells were plated in small culture flasks and were allowed to adhere overnight. Starting 10 min before BLI measurements, cells were incubated with a continuous flow of either 100% O_2_ or a mixture of different fractions of oxygen in N_2_ above the cell media. Quantification of the BLI signal intensity showed no significant differences (n = 4).
(TIF)

Figure S2 **Principles of unanesthetized imaging.** (**A**) “*in actio*” module that is inserted into the camera to enable the imaging of unanesthetized mice. (**B**) Representation of the module. The registration video is done using a near-infrared (NIR) camera and a near-infrared laser for illumination. A dichroic beam splitter allows direct transmission (with 95% efficiency) of the bioluminescence signal while reflecting the NIR light at an angel of 90°. (**C**) Principle of the dynamic fusion of video data and bioluminescence data. (**D**) The Laser and the intensified camera are switched on and off in opposition. When the laser is switch on, the intensifier camera is switched off and vice versa. The laser is switched on 1.5 ms every 22 ms, with a delay of 0.5 ms after the extinction of the laser. The intensified camera is then acquiring the bioluminescent signal during 20 ms every 22 ms. *CCD charge-coupled device. ICCD intensified CCD. IR infrared*. Figure from PhD thesis of Mickaël Savinaud entitled [Registration of the flux in kinematic data: application in optical imaging], in *French*.
(TIF)

Video S1 **Unanesthetized BLI.** Imaging in unanesthetized mice was achieved by using a dedicated system that allows the simultaneous recording of both bioluminescence signal and a bright field video of the animal under infrared illumination for co-registration. This video shows an example of a mouse bearing 293T-Fluc cells at the neck region after the injection of D-luciferin. For quantification, the same large region of interest covering the whole area of movement was used for both unanesthetized and anesthetized imaging.
(AVI)
