# Supplementary figures and images for: Inhibition of Firefly Luciferase by General Anesthetics: Effect on In Vitro and In Vivo Bioluminescence Imaging
Source: PLoS One. 2012 Jan 10;7(1):e30061. doi: 10.1371/journal.pone.0030061 (PMC3254645; doi:10.1371/journal.pone.0030061)

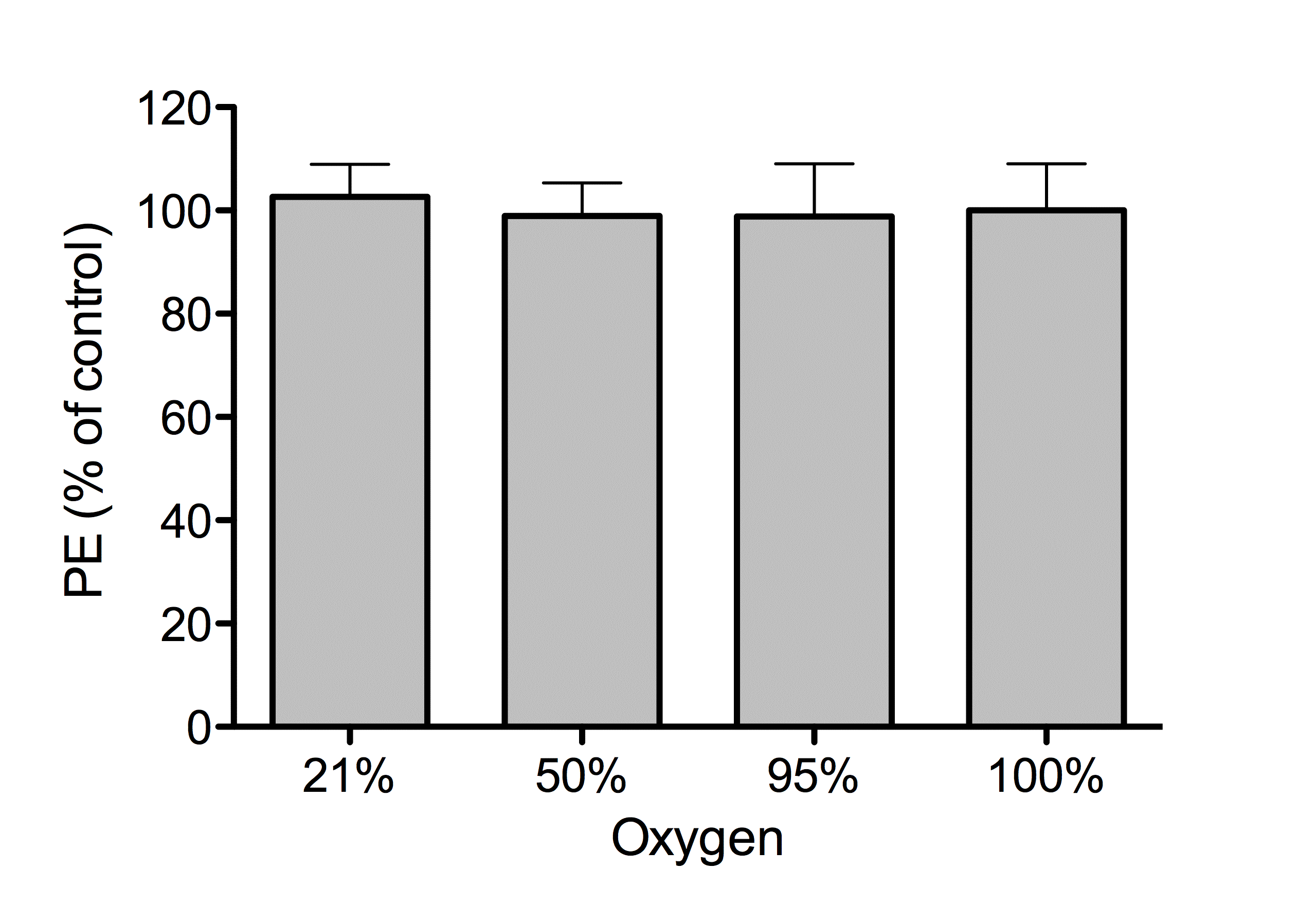

Supplement: Figure S1 — Comparison of BLI intensity using different fractions of oxygen. 1×10^6^ R1M-Fluc cells were plated in small culture flasks and were allowed to adhere overnight. Starting 10 min before BLI measurements, cells were incubated with a continuous flow of either 100% O2 or a mixture of different fractions of oxygen in N2 above the cell media. Quantification of the BLI signal intensity showed no significant differences (n = 4). (TIF) [file pone.0030061.s001.tif]

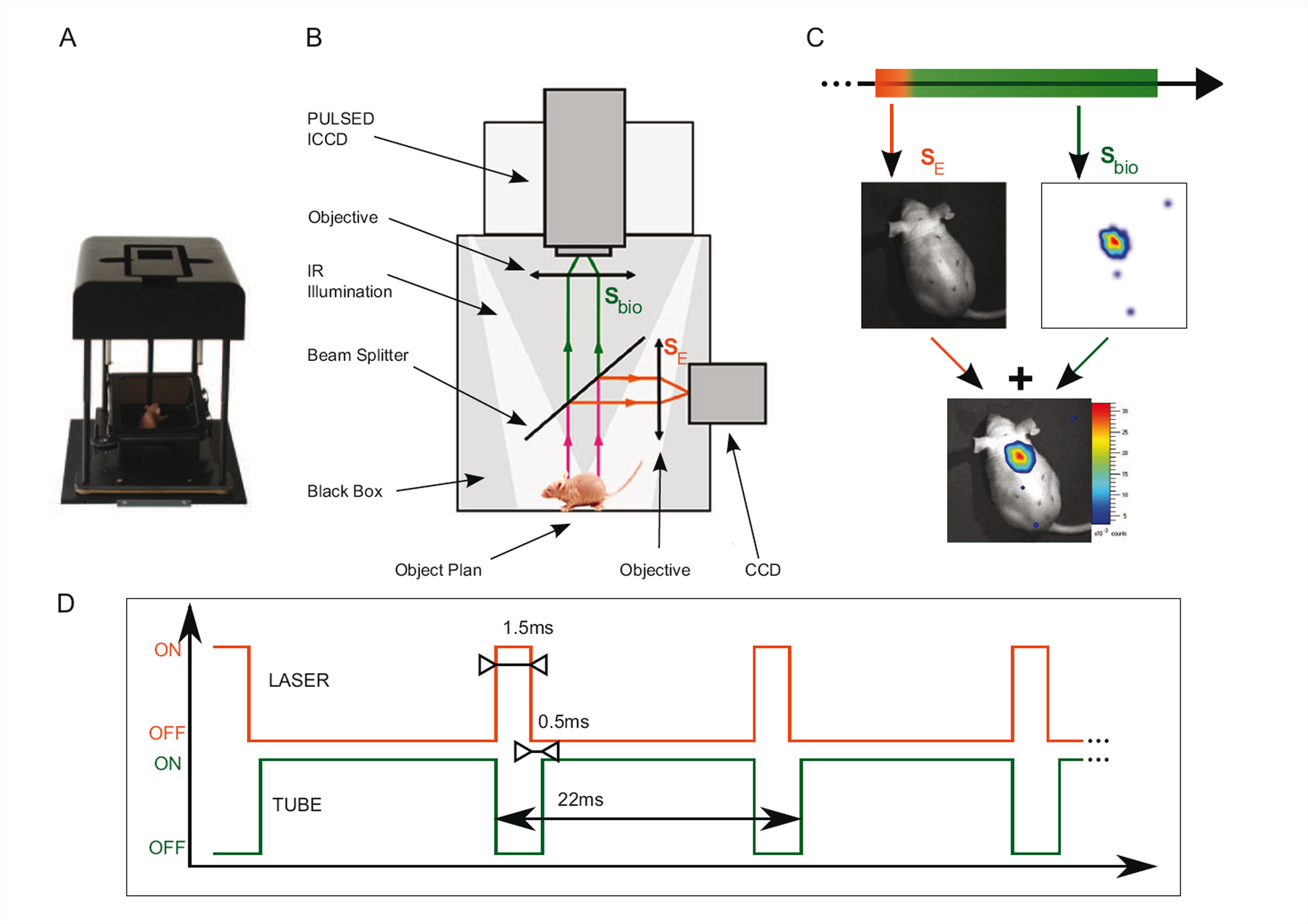

Supplement: Figure S2 — Principles of unanesthetized imaging. (A) “in actio” module that is inserted into the camera to enable the imaging of unanesthetized mice. (B) Representation of the module. The registration video is done using a near-infrared (NIR) camera and a near-infrared laser for illumination. A dichroic beam splitter allows direct transmission (with 95% efficiency) of the bioluminescence signal while reflecting the NIR light at an angel of 90°. (C) Principle of the dynamic fusion of video data and bioluminescence data. (D) The Laser and the intensified camera are switched on and off in opposition. When the laser is switch on, the intensifier camera is switched off and vice versa. The laser is switched on 1.5 ms every 22 ms, with a delay of 0.5 ms after the extinction of the laser. The intensified camera is then acquiring the bioluminescent signal during 20 ms every 22 ms. CCD charge-coupled device. ICCD intensified CCD. IR infrared. Figure from PhD thesis of Mickaël Savinaud entitled [Registration of the flux in kinematic data: application in optical imaging], in French. (TIF) [file pone.0030061.s002.tif]
